# Treatments During Pregnancy Targeting ERBB2 and Outcomes of Pregnant Individuals and Newborns

**DOI:** 10.1001/jamanetworkopen.2023.39934

**Published:** 2023-10-26

**Authors:** Paul Gougis, Beatriz Grandal, Floriane Jochum, Kevin Bihan, Florence Coussy, Solenn Barraud, Bernard Asselain, Elise Dumas, Clara Sebbag, Judicael Hotton, Emmanuel Spaggiari, Jean-Yves Pierga, Raphaëlle Savarino, Enora Laas, Jean-Philippe Spano, Fabien Reyal, Anne-Sophie Hamy

**Affiliations:** 1 Residual Tumor & Response to Treatment Laboratory, RT2Lab, Institut National de la Santé et de la Recherche Médicale, U932 Immunity and Cancer, Institut Curie, Université Paris Cité, Paris, France; 2 Department of Medical Oncology, Pitié-Salpêtrière Hospital, Paris, France, Assistance Publique–Hôpitaux de Paris, Paris, France; 3 Sorbonne Université, Institut National de la Santé et de la Recherche Médicale, Assistance Publique–Hôpitaux de Paris, Clinical Investigation Center (CIC-1901), Department of Pharmacology, Pitié-Salpêtrière Hospital, Paris, France; 4 Department of Gynecology, Strasbourg University Hospital, Strasbourg, France; 5 Department of Medical Oncology, Institut Curie, Université Paris Cité, Paris, France; 6 Department of Statistics, Association de Recherche sur les Cancers dont Gynécologiques–Groupe d’Investigateurs National des Etudes des Cancers Ovariens et du sein (ARCAGY-GINECO), Paris, France; 7 Department of Surgical Oncology, Institut Godinot, Reims, France; 8 Department of Obstetrics and Maternal-Fetal Medicine, Assistance Publique–Hôpitaux de Paris, Necker Enfants-Malades Hospital, Paris, France; 9 Department of Breast, Gynecological and Reconstructive Surgery, Institut Curie, Université Paris Cité, Paris, France; 10 Institut National de la Santé et de la Recherche Médicale, UMRS 1136, Paris, France

## Abstract

**Importance:**

Targeted therapies directed against ERBB2 are the cornerstone of medical treatment for ERBB2-positive breast cancers but are contraindicated during pregnancy.

**Objectives:**

To describe the association of exposure to anti-ERBB2 agents during pregnancy with pregnancy and fetal or newborn outcomes, and to compare the risk and types of adverse outcomes reported more frequently in this context than after exposure to other anticancer agents.

**Design, Setting, and Participants:**

For this case-control study, All reports with a pregnancy-related condition and an antineoplastic agent (Anatomical Therapeutic Chemical classification group L01) registered in the World Health Organization international pharmacovigilance database VigiBase up to June 26, 2022, were extracted. All reports with a pregnancy, an antineoplastic treatment during pregnancy, and a cancer were retained. Reports with anticancer agents prescribed for nononcologic purposes were not included.

**Exposure:**

The exposure group was defined as reports that mention anti-ERBB2 agents compared with exposure to other anticancer agents.

**Main Outcome and Measures:**

The main outcome was the reporting odds ratio (ROR) for maternofetal complications in the group exposed to anti-ERBB2 agents compared with other anticancer agents, as determined using a disproportionality analysis.

**Results:**

A total of 3558 reports (anti-ERBB2 agents, 328; other anticancer agents, 3230) were included in the analysis. In the group exposed to anti-ERBB2 agents, most reports were from the US (159 [48.5%]), the mean (SD) age of participants was 30.8 (10.4) years, and 209 patients (97.7%) were treated for breast cancers. The molecules most frequently involved in cases with anti-ERBB2 agents were trastuzumab (n = 302), pertuzumab (n = 55), trastuzumab-emtansine (n = 20), and lapatinib (n = 18). The outcomes overreported in these cases included oligohydramnios (ROR, 17.68 [95% CI, 12.26-25.52]; *P* < .001), congenital respiratory tract disorders (ROR, 9.98 [95% CI, 2.88-34.67]; *P* < .001), and neonatal kidney failure (ROR, 9.15 [95% CI, 4.62-18.12]; *P* < .001). Sensitivity and multivariable analyses found similar results. Toxic effects were also significantly overreported for trastuzumab-emtansine (cardiovascular malformation: ROR, 4.46 [95% CI, 1.02-19.52]) and lapatinib (intrauterine growth restriction: ROR, 7.68 [95% CI, 3.01-19.59]).

**Conclusions and Relevance:**

In this case-control study of 328 individuals exposed to anti-ERBB2 agents during pregnancy, exposure was associated with a severe specific adverse pregnancy and fetal or newborn outcomes compared with exposure to other anticancer treatments.

## Key Points

**Question:**

What pregnancy and fetal or newborn outcomes are associated with exposure to anti-ERBB2 agents during pregnancy?

**Findings:**

This case-control study with a disproportionality analysis was performed on 3558 reports from the World Health Organization international pharmacovigilance database that mention exposure to anticancer agents during pregnancy. Exposure to anti-ERBB2 agents during pregnancy (n = 328) was significantly associated with an overreporting of oligohydramnios, congenital respiratory tract disorders, and neonatal kidney failure relative to other anticancer drugs (n = 3230).

**Meaning:**

These findings suggest that anti-ERBB2 agents are associated with severe adverse fetal or newborn outcomes and should be contraindicated during pregnancy.

## Teaser

This case-control study describes the association of exposure to anti-ERBB2 agents during pregnancy with pregnancy and fetal or newborn outcomes compared with exposure to other anticancer agents.

## Introduction

Breast cancer (BC) is the most common cancer among young women and pregnant women.^1^ By 2022, anti-ERBB2 drugs had become the cornerstone of medical treatment for ERBB2-positive BCs, and their use during pregnancy, while rare, is expected to become more common. The management of BC among pregnant women is particularly challenging. Whenever possible, BCs occurring during pregnancy should be treated as recommended for nonpregnant patients.^2^ However, the benefits of treatment to the mother must be weighed against the potential harm to the fetus, due to congenital malformations and adverse obstetric or neonatal outcomes. In current guidelines,^2,3,4^ the use of trastuzumab during pregnancy is contraindicated, due principally to the risk of oligohydramnios and/or anhydramnios, conditions reported in 18 published case reports in this context,^5^ and to the unknown consequences for the fetus. The use of other anti-ERBB2 agents is also discouraged due to the absence of safety data obtained in the pregnancy setting. Given the major benefits associated with anti-ERBB2 therapies for ERBB2-positive BC, data from large-scale studies confirming or ruling out the toxic effects of these agents during pregnancy are crucial.

The World Health Organization VigiBase, a global pharmacovigilance database, contains more than 30 million individual case safety reports of adverse drug reactions occurring in more than 130 countries since 1967.^6^ This database has proved useful for the identification of new adverse drug reaction signals.^7,8^ In oncology,^9^ current guidelines recommend that (1) any pregnancy occurring during a trial or routine oncologic care should be monitored and that both (2) maternofetal adverse drug reactions and (3) outcomes of maternofetal exposure to contraindicated drugs, such as anticancer drugs, should be reported to pharmacovigilance institutions, including normal pregnancies, and ultimately reported to VigiBase. VigiBase was also previously investigated to address the fetal toxic effects of immune checkpoint inhibitors, for which no significant toxic effect was found.^10^

The objective of this study was to perform a large-scale descriptive analysis of pregnancy and fetal or newborn outcomes after exposure to anti-ERBB2 agents and to determine the reporting odds ratio (ROR) of adverse pregnancy and fetal outcomes after exposure to anti-ERBB2 drugs relative to other non–anti-ERBB2 anticancer drugs in a case-control disproportionality analysis.

## Methods

### Study Design

We performed a case-control disproportionality analysis study (also known as a case-noncase study), using pharmacovigilance reports from VigiBase, to evaluate the association of maternal and fetal or newborn adverse outcomes with exposure to anti-ERBB2 drugs compared with exposure to other anticancer drugs in reports of patients with cancer and anticancer drug exposure during pregnancy. The data in the VigiBase database are anonymized, and it is not possible to access personal information about the patients or the individuals reporting the cases. We adhered to all applicable legislation such as, but not limited to, EU and national legislation regarding the protection of personal data. The study followed the Strengthening the Reporting of Observational Studies in Epidemiology (STROBE) reporting guideline for case-control studies. The institutional review board of Institut Curie (Comité de Revue Institutionnelle–CRI Data) granted study approval.

### Data Query and Report Extraction

VigiBase was queried on June 26, 2022, with MedDRA, version 25.0. We extracted reports from VigiBase containing 1 or more pregnancy-related reactions and suspecting 1 or more anticancer drugs.

- Reactions associated with pregnancy were defined as any reaction with a reported term falling in the following MedDRA dictionary categories: pregnancy, puerperium and perinatal conditions (system organ classification); fetal and neonatal investigations (high-level group term [HLGT]); neonatal and perinatal conditions (HLGT); neonatal respiratory disorders (HLGT); exposures associated with pregnancy, delivery, and lactation (high-level term [HLT]); fetal therapeutic procedures (HLT); induced abortions (HLT); and obstetric therapeutic procedures (HLT). Details are summarized in eTable 1 in Supplement 1.
- A report was suspecting an anticancer drug when 1 or more anticancer drugs were notified as “suspect” (or “interacting”). Anticancer drugs were any drugs from the “antineoplasic” Anatomical Therapeutic Chemical (ATC) classification group L01.

### Data Cleaning and Exclusion Criteria

We ensured that only reports mentioning pregnancy-associated conditions or exposure were retained by discarding reports with terms secondarily associated with pregnancy. Only reports with terms primarily associated with pregnancy as a main system organ classification, HLGT, or HLT were retained. Reports were then analyzed to discard:

- Reports with no mention of a cancer diagnosis or with an antineoplastic drug from the L01 ATC group prescribed for a noncancer indication (eg, prescription of methotrexate for rheumatoid arthritis or of alemtuzumab for multiple sclerosis); and
- Reports with drug mapping problems or adverse event mapping problems (eTable 2 in Supplement 1).

### Modality of Exposure During Pregnancy

Timing and modality of anticancer drug exposure were identified using the preferred terms found in the reports (eTable 3 in Supplement 1). Exposure types were exposure before pregnancy, exposure during pregnancy, exposure via breast milk, exposure via semen, and exposure via skin. Reports with a biological sex indicated as male were considered to be exposed via semen. Reports with notification of a term associated with exposure via skin or semen were excluded. Reports with exposure via breast milk or before pregnancy and no specific mention of exposure during pregnancy were also discarded. All other reports were considered to have mentioned exposure to anticancer drugs during pregnancy.

### Definition of Exposure Groups

We considered the following US Food and Drug Administration (FDA)–approved drugs to be anti-ERBB2 drugs: anti-ERBB2 antibodies (trastuzumab and pertuzumab), anti-ERBB2 antibody-drug conjugates (trastuzumab-emtansine and trastuzumab-deruxtecan), and ERBB2 kinase inhibitors (lapatinib, tucatinib, and neratinib). Any report from the study analysis that mentioned an anti-ERBB2 drug was qualified as an anti-ERBB2 drug exposure group. Reports with other anticancer drugs and no mention of anti-ERBB2 drugs were qualified as an exposure to other anticancer drugs group.

### Definition of Cases and Controls for Maternal and/or Fetal or Newborn Outcomes

Noncases within this disproportionality analysis are referred to as controls. Adverse events of interest were maternal and fetal or newborn adverse outcomes mapped directly to MedDRA-preferred terms in VigiBase. They constituted 30 individual maternal-fetal adverse outcomes regrouped into 7 categories for the purposes of this study:

- Abortions: induced abortion and spontaneous abortion;
- Stillbirth: fetal death;
- Congenital malformation: cardiovascular malformation, fetal malformation not otherwise specified (NOS), congenital respiratory tract disorders, genetic disorders, musculoskeletal malformation, sensory defect, neurologic malformation, and genitourinary malformation;
- Pregnancy complications: oligohydramnios, intrauterine growth restriction, pregnancy complication NOS, gestational hypertension and preeclampsia, polyhydramnios, gestational diabetes, and pregnancy hemorrhage;
- Preterm birth;
- Neonatal complications: neonatal respiratory disorder, neonatal kidney failure, neonatal infection, neonatal neuronal disorder, neonatal hematologic disorder, neonatal digestive disorder, neonatal cardiovascular disorder, neonatal effusion, hyperbilirubinemia, and neonatal metabolic-endocrine disorder; and
- Delivery complication.

Some adverse outcomes were deemed not clinically relevant (eg, somatic symptoms of pregnancy, lactation disorder, “large-for-date baby,” and “postmature baby”) and were discarded (eTable 4 in Supplement 1). For the independent analysis of each adverse event, cases were defined as study population reports that mentioned an adverse event. Controls were defined as all other study reports with no mention of an adverse event.

### Mitigation of Biases and Confounding Factors

To assess the robustness of our results, we conducted 3 sensitivity or subgroup analyses. First, we analyzed the ROR of each toxic effect within the subpopulation with BC. Second, we analyzed reports for which a single class of treatment was used (anti-ERBB2 drugs in the anti-ERBB2 drug exposure group). For this analysis, any report with a combination of drug classes, such as cytotoxic plus anti-ERBB2 drugs, was discarded. Third, we conducted a disproportionality analysis for each anti-ERBB2 molecule independently.

To limit the possibility of biases, we also identified confounding variables using a directed acyclic graph (eFigure 1 in Supplement 1). We identified the year of the report, the country of the report, individual’s age, and the cancer type as the main variables that need to be adjusted to limit confounding factors. The odds ratio for the risk of each toxic effect was then evaluated using a multivariable analysis by logistic regression with adjustment on these variables. Missing data were grouped within a single level of value for each variable.

### Statistical Analysis

We performed a case-control study, using a disproportionality analysis to evaluate the association between an adverse outcome of interest and an exposure. The ROR was defined as the ratio of the odds of the adverse pregnancy or fetal or newborn outcome of interest with exposure to anti-ERBB2 drugs to the odds with exposure to other anticancer drugs (contingency table in eTable 5 in Supplement 1): ROR = (*a*/*c*)/(*b*/*d*) = *ad*/*bc*.

A signal was considered to be present when statistically significant disproportionality was demonstrated between cases and controls. The interpretation rules were as follows^11^:

- If the ROR is 1, there is no signal; the outcome of interest was equally frequent in exposed and nonexposed reports;
- If the ROR is less than 1, there is no signal; the outcome of interest was less frequent in exposed reports (anti-ERBB2 drugs) compared with nonexposed reports (other anticancer drugs), and no firm conclusion could be drawn;
- If the ROR is greater than 1, the outcome of interest was more frequent in exposed reports (anti-ERBB2 drugs) compared with nonexposed reports (other anticancer drugs); there is, thus, a pharmacovigilance signal, and the higher the ROR, the greater the disproportionality. The signal was considered to indicate significant overreporting among exposed reports when the ROR025, corresponding to the lower limit of the ROR 95% CI, was greater than 1.

The study population is described in terms of frequencies for qualitative variables or of median (IQR) values for quantitative variables. Associations between categorical variables were assessed using the χ^2^ test or the Fisher exact test if at least 1 category included fewer than 5 patients. All *P* values were from 1-sided tests and results were deemed statistically significant at *P* < .05.

## Results

### Report Characteristics

**Figure 1.**
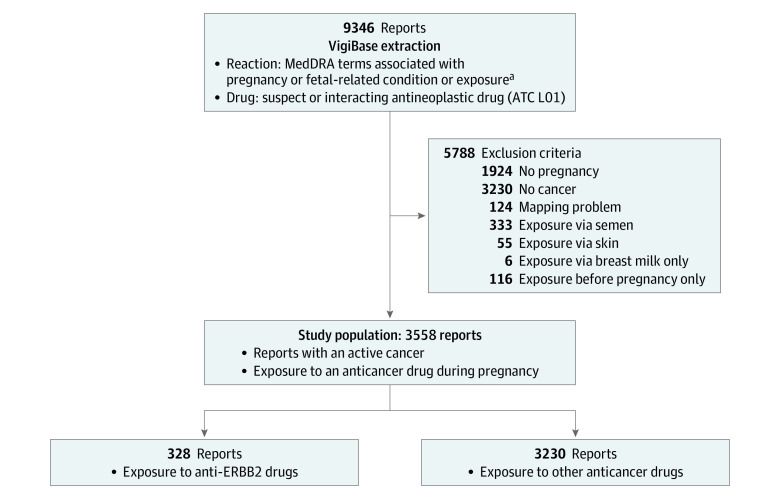
Study Flowchart All reports were extracted from VigiBase. Details of data extraction are provided in eTable 1 in Supplement 1. Terms associated secondarily with this request were not specific to pregnancy and were discarded. Reports also mentioned a suspect or interacting drug from the Anatomical Therapeutic Chemical (ATC) L01 class (antineoplastic drugs), which could have been prescribed for a cancer or noncancer indication. ^a^MedDRA terms queried for “Reaction” were pregnancy, puerperium, and perinatal conditions (system organ classifications); fetal and neonatal investigations (high-level group term [HLGT]); neonatal and perinatal conditions (HLGT); neonatal respiratory disorders (HLGT); exposures associated with pregnancy, delivery, and lactation (high-level term [HLT]); fetal therapeutic procedures (HLT); induced abortions (HLT); and obstetric therapeutic procedures (HLT).

We extracted 9346 deduplicated reports and retained 3558 reports for the final analysis (Figure 1) (anti-ERBB2 drug exposure, 328; other anticancer drugs, 3230). In the group exposed to anti-ERBB2 agents, most reports were from the US (159 [48.5%]), and the mean (SD) age 30.8 (10.4) years. Breast cancer was the cancer most frequently diagnosed in the anti-ERBB2 drug group (209 [97.7%]), whereas chronic myeloid leukemia (611 [29.6%]) and BC (476 [23.1%]) were the 2 most frequent types of cancer in the other anticancer drug group (Table 1; eFigure 2 in Supplement 1). No reports with exposure to trastuzumab-deruxtecan or neratinib were found, and only 1 report of tucatinib exposure was found. The earliest record identified in the anti-ERBB2 drug exposure group was from 2002, and the earliest record identified for other anticancer drugs was from 1978. However, there were significantly more reports in 2009 or before with exposure to anti-ERBB2 drugs (48 [14.6%]) compared with other anticancer drugs (314 [9.7%]; *P* < .001) (Table 1).

**Table 1.** Characteristics of Reports in the Study Population and Detail for Reports With Exposure to Anti-ERBB2 Drugs and Other Anticancer Drugs^a^

Variable	No. (%)			*P* value
	Exposure to anti-ERBB2 drugs (n = 328 [9.2%])	Exposure to other anticancer drugs (n = 3230 [90.8%])	Total (N = 3558)	
Age, mean (SD), y	30.8 (10.4)	28.5 (12.5)	28.7 (12.4)	.04
Country group				
Africa	11 (3.4)	102 (3.2)	113 (3.2)	<.001
America, other than US	20 (6.1)	110 (3.4)	130 (3.7)	
Asia	18 (5.5)	555 (17.2)	573 (16.1)	
Europe, other than Germany	82 (25.0)	681 (21.1)	763 (21.4)	
Germany	26 (7.9)	168 (5.2)	194 (5.5)	
Oceania	12 (3.7)	60 (1.9)	72 (2.0)	
US	159 (48.5)	1554 (48.1)	1713 (48.1)	
Notifier type				
Consumer or nonhealth professional	38 (12.6)	305 (10.1)	343 (10.4)	<.001
Other health professional	73 (24.2)	1155 (38.3)	1228 (37.1)	
Physician or pharmacist	191 (63.2)	1552 (51.5)	1743 (52.6)	
Year of first report				
2009 or before	48 (14.6)	314 (9.7)	362 (10.2)	<.001
2010-2014	57 (17.4)	861 (26.7)	918 (25.8)	
2015-2019	157 (47.9)	1454 (45.0)	1611 (45.3)	
2020-2022	66 (20.1)	601 (18.6)	667 (18.7)	
Number of suspect or interacting drugs				
1	159 (48.5)	1357 (42.0)	1516 (42.6)	<.001
2	74 (22.6)	540 (16.7)	614 (17.3)	
≥3	95 (29.0)	1333 (41.3)	1428 (40.1)	
Clinical setting				
Clinical trial	22 (6.7)	84 (2.6)	106 (3.0)	<.001
Routine care	306 (93.3)	3146 (97.4)	3452 (97.0)	
Cancer subtype				
Breast	209 (97.7)	476 (23.1)	685 (30.1)	<.001
Gastroesophageal	2 (0.9)	8 (0.4)	10 (0.4)	
Lung	3 (1.4)	39 (1.9)	42 (1.8)	
Acute leukemia	0	182 (8.8)	182 (8.0)	
Chronic myeloid leukemia	0	611 (29.6)	611 (26.9)	
Other cancers	0	745 (36.1)	745 (32.7)	
Suspect molecule class				
Cytotoxic	133 (40.5)	1902 (58.9)	2035 (57.2)	<.001
Molecular targeted therapy	328 (100.0)	1561 (48.3)	1889 (53.1)	<.001
Immunotherapy	1 (0.3)	102 (3.2)	103 (2.9)	.006
Hormone therapy	35 (10.7)	40 (1.2)	75 (2.1)	<.001
Other or NOS anticancer agent	0	15 (0.5)	15 (0.4)	.43
Comedication	79 (24.1)	1002 (31.0)	1081 (30.4)	.01

Abbreviation: NOS, not otherwise specified.

^a^ Missing values: for anti-ERBB2 drug–exposed, missing data: age, 198; notifier type, 26; number of notifiers, 26; and number of cancers, 114. For other anticancer drug–exposed: age, 1836; notifier type, 218; number of notifiers, 218; and number of cancers, 1169.

### Exposure to Anticancer Drugs

More than half the reports in the anti-ERBB2 drug–exposed group involved anti-ERBB2 therapies only (182 of 328 [55.5%]), either in monotherapy (trastuzumab, 138; trastuzumab-emtansine, 12; lapatinib, 2; tucatinib, 1; pertuzumab, 1) or in combination (trastuzumab plus pertuzumab, 24; other anti-ERBB2 drug combinations, 4) (eFigure 3 in Supplement 1). The other anti-ERBB2 drug–exposed reports (147 of 328 [44.8%]) included combinations of anti-ERBB2 therapies with other anticancer agents (cytotoxic chemotherapy, 133; hormone therapy, 35; other molecular targeted therapies, 7; immunotherapy, 1). For the exposure to other anticancer drugs group, 1902 reports (58.9%) mentioned at least 1 administration of cytotoxic chemotherapy, 1561 (48.3%) mentioned a molecular targeted therapy, and 157 (4.9%) mentioned other drug classes (Table 1).

### Pregnancy and/or Fetal or Newborn Outcomes

**Figure 2.**
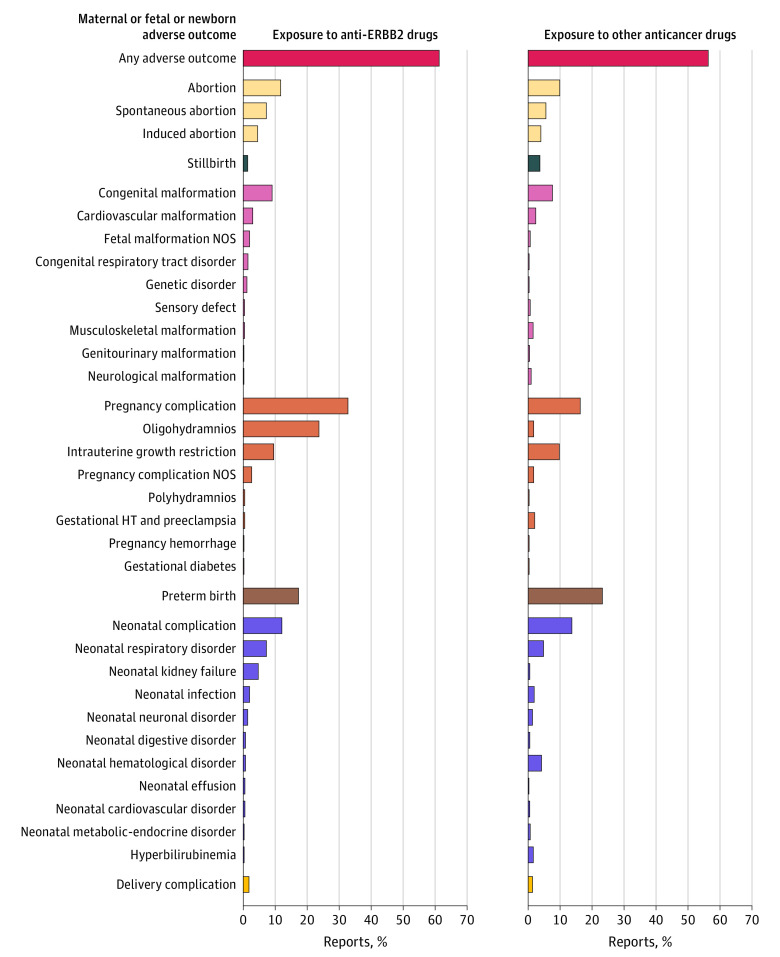
Description of Pregnancy and Fetal or Newborn Adverse Outcomes With Exposure to Anti-ERBB2 Drugs Compared With Exposure to Other Anticancer Drugs Bars represent the percentage of each outcome divided by the total number of reports. Counts are also annotated. For congenital respiratory tract disorders, the anti-ERBB2 drug–exposed group included 2 reports of pulmonary hypoplasia, 2 reports of respiratory tract malformation, and 1 report of laryngomalacia. The exposure to other anticancer drugs group included 3 reports of diaphragmatic hernia and 2 reports of pulmonary hypoplasia. HT indicates hypertension; NOS, not otherwise specified.

**Figure 3.**
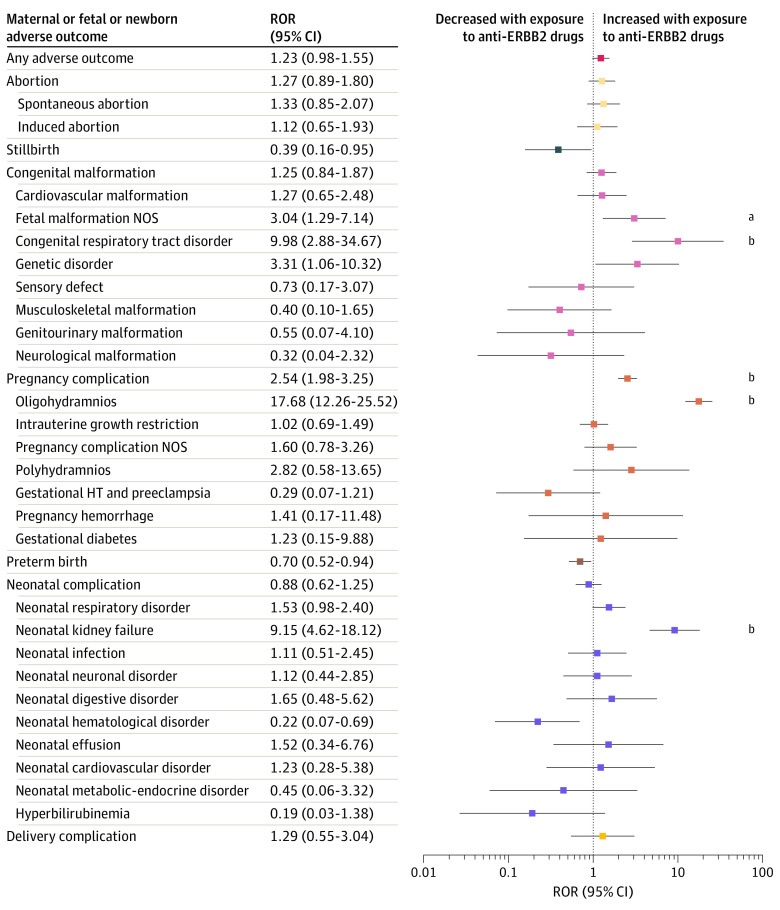
Reporting Odds Ratios (RORs) of Pregnancy and Fetal or Newborn Adverse Outcomes With Exposure to Anti-ERBB2 Drugs Compared With Exposure to Other Anticancer Drugs RORs are displayed as logarithmic data for the purpose of data visualization. HT indicates hypertension; NOS, not otherwise specified. ^a^*P* ≤ .05. ^b^*P* ≤ .001.

Pregnancy or fetal or newborn adverse outcomes were reported in 201 reports in the anti-ERBB2 drug group (61.3%) and in 1817 reports (56.3%) in the other anticancer drug group (Figure 2). The 5 complications most frequently reported in the anti-ERBB2 drug group were oligohydramnios (78 [23.8%]), preterm birth (57 [17.4%]), intrauterine growth restriction (32 [9.8%]), neonatal respiratory disorder (24 [7.3%]), and spontaneous abortion 24 [7.3%]). Complications were generally isolated (eFigure 4 in Supplement 1). Five neonates had neonatal respiratory disorders combined with kidney failure. The ROR was significantly higher than 1 in anti-ERBB2 drug–exposed reports for oligohydramnios (ROR, 17.68 [95% CI, 12.26-25.52]; *P* < .001), fetal malformation NOS (ROR, 3.04 [95% CI, 1.29-7.14]; *P* = .02), congenital respiratory tract disorders (ROR, 9.98 [95% CI, 2.88-34.67]; *P* = .001), and neonatal kidney failure (ROR, 9.15 [95% CI, 4.62-18.12]; *P* < .001) (Figure 3).

Similar results were obtained for trastuzumab and pertuzumab in a subgroup analysis by type of anti-ERBB2 molecule (Table 2; eFigure 5 in Supplement 1). In reports that mentioned exposure to pertuzumab, the ROR was significantly higher than 1 for congenital respiratory tract disorder (ROR, 16.49 [95% CI, 3.42-79.49]; *P* < .001) and oligohydramnios (ROR, 4.56 [95% CI, 2.11-9.86]; *P* < .001). There was an overreporting of cardiovascular malformation (ROR, 4.46 [95% CI, 1.02-19.52]) with trastuzumab-emtansine exposure (n = 20), although the Fisher exact test did not reach statistical significance (*P* = .09). Only intrauterine growth restriction was overreported (ROR, 7.68 [95% CI, 3.01-19.59]) after lapatinib exposure (n = 18).

**Table 2.** Number of Counts for Cases Compared With Controls and Corresponding Reporting Odds Ratios for Each Pregnancy and/or Fetal or Newborn Outcome for Each Anti-ERBB2 Molecule Individually

Pregnancy or fetal or newborn outcome	Trastuzumab (n = 302)			Pertuzumab (n = 55)			Trastuzumab-emtansine (n = 20)			Lapatinib (n = 18)		
	No. (%)	ROR (95% CI)	*P* value	No. (%)	ROR (95% CI)	*P* value	No. (%)	ROR (95% CI)	*P* value	No. (%)	ROR (95% CI)	*P* value
Any adverse outcome	186 (61.6)	1.25 (0.98-1.59)	.08^a^	25 (45.5)	0.63 (0.37-1.08)	.10	10 (50)	0.76 (0.32-1.84)	.65	12 (66.7)	1.53 (0.57-4.08)	.48
Abortion	37 (12.3)	1.31 (0.91-1.89)	.16	5 (9.1)	0.92 (0.36-2.31)	≥.99	2 (10)	1.02 (0.24-4.41)	≥.99	1 (5.6)	0.54 (0.07-4.05)	≥.99
Spontaneous abortion	23 (7.6)	1.39 (0.89-2.19)	.15	4 (7.3)	1.29 (0.46-3.60)	.56	1 (5)	0.86 (0.11-6.46)	≥.99	0	NA	NA
Induced abortion	14 (4.6)	1.13 (0.64-1.99)	.65	1 (1.8)	0.42 (0.06-3.08)	.73	1 (5)	1.21 (0.16-9.13)	.57	1 (5.6)	1.36 (0.18-10.27)	.54
Stillbirth	4 (1.3)	0.34 (0.12-0.92)	.02^a^	0	NA	NA	0	NA	NA	1 (5.6)	1.57 (0.21-11.87)	.49
Congenital malformation	27 (8.9)	1.22 (0.80-1.85)	.36	5 (9.1)	1.22 (0.48-3.09)	.61	2 (10)	1.36 (0.31-5.87)	.66	1 (5.6)	0.72 (0.09-5.40)	≥.99
Cardiovascular malformation	9 (3)	1.24 (0.61-2.49)	.56	3 (5.5)	2.32 (0.71-7.58)	.15	2 (10)^a^	4.46 (1.02-19.52)^a^	.09^a^	0	NA	NA
Fetal malformation NOS	6 (2)^a^	2.73 (1.11-6.73)^a^	.04^a^	0	NA	NA	0	NA	NA	0	NA	NA
Congenital respiratory tract malformation	5 (1.7)^a^	10.95 (3.15-38.03)^a^	<.001^a^	2 (3.6)^a^	16.49 (3.42-79.49)^a^	<.001^a^	0	NA	NA	0	NA	NA
Genetic disorder	4 (1.3)^a^	3.63 (1.16-11.32)^a^	.04^a^	1 (1.8)	4.31 (0.56-33.18)	.22	0	NA	NA	0	NA	NA
Musculoskeletal malformation	2 (0.7)	0.21 (0.03-1.55)	≥.99	0	NA	NA	0	NA	NA	1 (5.6)	4.11 (0.54-31.45)	.23
Sensory defect	1 (0.3)	0.80 (0.19-3.37)	.12	0	NA	NA	0	NA	NA	0	NA	NA
Neurologic malformation	1 (0.3)	0.35 (0.05-2.54)	.52	0	NA	NA	0	NA	NA	0	NA	NA
Genitourinary malformation	1 (0.3)	0.60 (0.08-4.49)	≥.99	1 (1.8)	3.59 (0.47-27.34)	.26	0	NA	NA	0	NA	NA
Pregnancy complication	102 (33.8)^a^	2.62 (2.03-3.39)^a^	<.001^a^	14 (25.5)	1.59 (0.86-2.94)	.15	2 (10)	0.51 (0.12-2.22)	.56	8 (44.4)^a^	3.74 (1.47-9.51)^a^	.008^a^
Oligohydramnios	77 (25.5)^a^	19.21 (13.29-27.76)^a^	<.001^a^	8 (14.5)^a^	4.56 (2.11-9.86)^a^	<.001^a^	1 (5)	1.35 (0.18-10.14)	.54	0	NA	NA
Intrauterine growth restriction	28 (9.3)	0.96 (0.64-1.44)	.92	5 (9.1)	0.94 (0.37-2.37)	≥.99	0	NA	NA	8 (44.4)^a^	7.68 (3.01-19.59)^a^	<.001^a^
Pregnancy complication NOS	8 (2.6)	1.53 (0.72-3.23)	.26	0	NA	NA	0	NA	NA	1 (5.6)	3.19 (0.42-24.37)	.28
Gestational HT and preeclampsia	1 (0.3)^a^	0.16 (0.02-1.14)	.17	1 (1.8)	0.95 (0.13-6.97)	≥.99	1 (5)	2.73 (0.36-20.67)	.32	0	NA	NA
Polyhydramnios	2 (0.7)	3.09 (0.64-14.96)^a^	.03^a^	0	NA	NA	0	NA	NA	0	NA	NA
Gestational diabetes	1 (0.3)	1.35 (0.17-10.82)	.55	0	NA	NA	0	NA	NA	0	NA	NA
Pregnancy hemorrhage	1 (0.3)	1.54 (0.19-12.58)	.51	0	NA	NA	0	NA	NA	0	NA	NA
Preterm birth	52 (17.2)^a^	0.69 (0.51-0.94)^a^	.02^a^	7 (12.7)	0.49 (0.22-1.10)	.10	3 (15)	0.60 (0.18-2.06)	.59	6 (33.3)	1.72 (0.64-4.58)	.27
Neonatal complication	39 (12.9)	0.95 (0.67-1.35)	.86	3 (5.5)	0.37 (0.11-1.18)	.11	2 (10)	0.71 (0.16-3.08)	≥.99	0	NA	NA
Neonatal respiratory disorder	24 (7.9)^a^	1.69 (1.08-2.65)^a^	.03^a^	0	NA	NA	0	NA	NA	0	NA	NA
Neonatal kidney failure	16 (5.3)^a^	10.06 (5.08-19.95)^a^	<.001^a^	2 (3.6)	4.09 (0.96-17.52)	.10	0	NA	NA	0	NA	NA
Neonatal infection	7 (2.3)	1.22 (0.55-2.69)	.66	1 (1.8)	0.94 (0.13-6.86)	≥.99	1 (5)	2.69 (0.35-20.35)	.32	0	NA	NA
Neonatal neuronal disorder	5 (1.7)	1.23 (0.48-3.12)	.60	0	NA	NA	0	NA	NA	0	NA	NA
Neonatal hematologic disorder	3 (1)^a^	0.24 (0.08-0.76)^a^	.004^a^	0	NA	NA	0	NA	NA	0	NA	NA
Neonatal digestive disorder	3 (1)	1.80 (0.53-6.16)	.42	0	NA	NA	0	NA	NA	0	NA	NA
Neonatal cardiovascular disorder	2 (0.7)	1.35 (0.31-5.90)	.66	0	NA	NA	0	NA	NA	0	NA	NA
Neonatal effusion	2 (0.7)	1.66 (0.37-7.4)	.37	0	NA	NA	0	NA	NA	0	NA	NA
Hyperbilirubinemia	0	NA	NA	0	NA	NA	1 (5)	3.60 (0.47-27.4)	.26	0	NA	NA
Neonatal metabolic-endocrine disorder	1 (0.3)	0.49 (0.07-3.64)	.25	0	NA	NA	0	NA	NA	0	NA	NA
Delivery complication	6 (2)	1.41 (0.60-3.34)	.45	0	NA	NA	0	NA	NA	0	NA	NA

Abbreviations: HT, hypertension; NA, not applicable; NOS, not otherwise specified; ROR, reporting odds ratio.

^a^ Outcomes with *P* < .05 and/or when the ROR025, corresponding to the lower limit of the ROR 95% CI, was greater than 1.

### Sensitivity and Multivariable Analyses

In multivariable analysis (eFigure 6 in Supplement 1) after adjustment for the year and country of the report, individual’s age, and tumor type, main toxicity signals remained: congenital respiratory tract disorder (odds ratio [OR], 14.0 [95% CI, 3.2-69.8]; *P* < .001), oligohydramnios (OR, 19.0 [95% CI, 11.7-31.5]; *P* < .001), and neonatal or fetal kidney failure (OR, 9.6 [95% CI, 4.2-22.4]; *P* < .001). The details of the multivariable analysis are available in eTable 6 in Supplement 1. Spontaneous abortion was also significantly increased (OR, 2.5 [95% CI, 1.4-4.2]; *P* < .001) as well as neonatal respiratory disorders (OR, 1.8 [95% CI, 1.1-2.9]; *P* = .02) (eFigure 6 in Supplement 1). Sensitivity analyses performed on the subpopulation treated for BC only, treated with anti-ERBB2 drugs only, and with an explicit exposure declaration identified similar pregnancy and/or fetal or newborn outcome profiles, with a significant overreporting of oligohydramnios and neonatal kidney failure (eFigures 7 and 8 in Supplement 1).

## Discussion

In this study, we analyzed what is, to our knowledge, the largest reported series of cases of maternofetal exposure to anti-ERBB2 therapy during pregnancy. We found that specific adverse outcomes (oligohydramnios, congenital respiratory disorders, and neonatal kidney failure) were more frequently reported for anti-ERBB2 treatments than for other anticancer drugs.

First, the proportion of adverse pregnancy and fetal or newborn outcomes after anti-ERBB2 drug exposure during pregnancy was similar to that reported for exposure to other anticancer drugs (61.3% vs 56.3%; *P* = .09). In 28 studies, Andrikopoulou and coworkers^5^ identified 30 cases of patients (32 fetuses) exposed to trastuzumab during pregnancy; completely healthy neonates were born in only 43% of cases. In our study, we identified 78 reports of oligohydramnios among patients exposed to anti-ERBB2 agents during pregnancy (23.8%).^5,12,13^ This result confirms, at a larger scale, previous findings^5^ of 18 cases of oligohydramnios in 30 pregnancies (60.0%).

Second, we identified strong, highly significant overreporting of congenital respiratory tract disorders and neonatal kidney failure. These results confirm the signal reported by previous authors,^5^ who described 1 case of kidney failure and 1 case of pulmonary hypoplasia. ERBB2 expression plays an important, but as yet uncharacterized, role in the kidney during fetal development in rats and in the skin, lung, and intestine.^14^ Fetal kidney failure, leading to oligohydramnios, and subsequently to pulmonary hypoplasia and neonatal respiratory disorders, has been described in a Potter sequence.^15^

Third, we provide data for exposure to the new anti-ERBB2 agents pertuzumab, trastuzumab-emtansine, and lapatinib during pregnancy. Previous recommendations for these drugs were formulated on the basis of animal studies in the absence of comparative clinical data. Exposure to anti-ERBB2 agents other than trastuzumab-emtansine during pregnancy was not associated with an increase in cardiovascular neonatal complications or malformations in our study. This finding is reassuring because ERBB2 plays an important role in embryonic cardiac development^16^ and has been implicated in cardiac toxic effects in adults.^17^ However, the toxicity profiles of lapatinib and trastuzumab-emtansine were different from that of trastuzumab, and these drugs could be more harmful to fetuses, although more data are required to confirm these findings (Table 2; eFigure 5 in Supplement 1).

### Limitations

This study has some limitations; the main limitation of the pharmacovigilance approach is inconsistencies in the collection of information. This could limit the conclusions being drawn about the incidence of these events in the general population. Also, most reports had limited information about the exact timing of exposure, which limits the conclusion about the association of the duration of fetal exposition with outcomes. It could also be argued that the group exposed to other anticancer drugs constitutes a population different from that of the anti-ERBB2 drug–exposed group, which also limits the conclusions that can be drawn. Despite FDA contraindication and general knowledge that trastuzumab could have an adverse effect on pregnancies, we found several reports with oligohydramnios and anti-ERBB2 drug exposure into the second and third trimesters, allowing us to reject the violation of the positivity hypothesis. However, sensitivity analyses carried out among more homogenous subgroups, along with multivariable analyses adjusted for key variables, consistently reaffirmed our primary findings. More generally, the large sample sizes within the database facilitate the detection of signals and the comparative analysis of extremely rare events. This makes VigiBase a valuable resource for investigating intrauterine exposure to anticancer agents, but despite efforts to mitigate biases, the inherent variability in the quality of the data may constrain the conclusiveness of the findings.^11,18^

## Conclusions

The findings of our case-control study present substantial evidence that anti-ERBB2 therapies are associated with the occurrence of kidney failure as well as oligohydramnios among fetuses or newborns. These conditions may further escalate into respiratory tract malformation, which includes lung aplasia. Nevertheless, delaying the mother’s treatment might lead to severe outcomes, particularly in cases of hormone receptor–negative BCs. Furthermore, our research indicates that oligohydramnios may be associated with subsequent fetal and neonatal complications. Whenever delaying anti-ERBB2 therapy is not possible, continuous monitoring for oligohydramnios becomes imperative, and anti-ERBB2 drugs should be withdrawn in the case of oligohydramnios. This approach demands a thorough investigation to ascertain its safety, and dedicated research must be pursued to confirm the validity of this strategy. Our study also has revealed distinct pharmacologic and fetal toxicity profiles for lapatinib and trastuzumab-emtansine, leading us to advise against the use of these specific treatments in all pregnant individuals.

## Notes

Supplement 1. **eTable 1.** Detail of VigiBase Query **eTable 2.** Terms Corrected **eTable 3.** MedDRA Preferred Terms Used to Qualify Reports’ Exposure Type **eTable 4.** Terms Deemed Not Clinically Significant **eTable 5.** Contingency Table **eTable 6.** Details of Multivariable Analysis and Odds Ratio for Other Parameters for the Risk of “Oligohydramnios” **eFigure 1.** Directed Acyclic Graph for Assessment of Confounding Factors **eFigure 2.** Characteristics of Reports in Study Population **eFigure 3.** UpSet Plot of the Reporting of Anticancer Drugs in the Anti-ERBB2-Exposed Group **eFigure 4.** UpSet Plot of the Co-reporting of Adverse Pregnancy and Fetal/Newborn Outcomes Overreported in Cases Exposed to Anti-ERBB2 **eFigure 5.** Disproportionality Analysis for Each Molecule **eFigure 6.** Multivariable Analysis of the Risk of Adverse Outcomes **eFigure 7.** Sensitivity Analysis Within the Subpopulation of Reports With an Identified Breast Cancer **eFigure 8.** Sensitivity Analysis Within the Subpopulation of Reports Treated With Single-Class Drugs

Supplement 2.
Data Sharing Statement
