# Supplementary material for: Treatments During Pregnancy Targeting ERBB2 and Outcomes of Pregnant Individuals and Newborns
Source: JAMA Netw Open. 2023 Oct 26;6(10):e2339934. doi: 10.1001/jamanetworkopen.2023.39934 (PMC10603505; doi:10.1001/jamanetworkopen.2023.39934)
Supplement: Supplement 1. — eTable 1. Detail of VigiBase Query eTable 2. Terms Corrected eTable 3. MedDRA Preferred Terms Used to Qualify Reports’ Exposure Type eTable 4. Terms Deemed Not Clinically Significant eTable 5. Contingency Table eTable 6. Details of Multivariable Analysis and Odds Ratio for Other Parameters for the Risk of “Oligohydramnios” eFigure 1. Directed Acyclic Graph for Assessment of Confounding Factors eFigure 2. Characteristics of Reports in Study Population eFigure 3. UpSet Plot of the Reporting of Anticancer Drugs in the Anti-ERBB2-Exposed Group eFigure 4. UpSet Plot of the Co-reporting of Adverse Pregnancy and Fetal/Newborn Outcomes Overreported in Cases Exposed to Anti-ERBB2 eFigure 5. Disproportionality Analysis for Each Molecule eFigure 6. Multivariable Analysis of the Risk of Adverse Outcomes eFigure 7. Sensitivity Analysis Within the Subpopulation of Reports With an Identified Breast Cancer eFigure 8. Sensitivity Analysis Within the Subpopulation of Reports Treated With Single-Class Drugs [file jamanetwopen-e2339934-s001.pdf]

Supplementary Online Content

Gougis P, Grandal B, Jochum F, et al. Treatments during pregnancy targeting ERBB2 and outcomes of pregnant individuals and newborns. *JAMA Netw Open.* 2023;6(10):e2339934. doi:10.1001/jamanetworkopen.2023.39934

eTable 1. Detail of VigiBase Query
eTable 2. Terms Corrected
eTable 3. MedDRA Preferred Terms Used to Qualify Reports' Exposure Type
eTable 4. Terms Deemed Not Clinically Significant
eTable 5. Contingency Table
eTable 6. Details of Multivariable Analysis and Odds Ratio for Other Parameters for the Risk of "Oligohydramnios"
eFigure 1. Directed Acyclic Graph for Assessment of Confounding Factors
eFigure 2. Characteristics of Reports in Study Population
eFigure 3. UpSet Plot of the Reporting of Anticancer Drugs in the Anti-ERBB2-Exposed Group
eFigure 4. UpSet Plot of the Co-reporting of Adverse Pregnancy and Fetal/Newborn Outcomes Overreported in Cases Exposed to Anti-ERBB2
eFigure 5. Disproportionality Analysis for Each Molecule
eFigure 6. Multivariable Analysis of the Risk of Adverse Outcomes
eFigure 7. Sensitivity Analysis Within the Subpopulation of Reports With an Identified Breast Cancer
eFigure 8. Sensitivity Analysis Within the Subpopulation of Reports Treated With Single-Class Drugs

This supplementary material has been provided by the authors to give readers additional information about their work.

© 2023 Gougis P et al. *JAMA Network Open.*

eTable 1. Detail of VigiBase Query

**VigiBase data set date**	06/26/2022	
**MedDRA version**	MedDRA 25.0 (English)	
**Search criteria for VigiBase extraction**	**drugs (ATC group)**	L01 ANTINEOPLASTIC AGENTS
	**reaction and MedDRA terms**	Pregnancy, puerperium and perinatal conditions (SOC)<br>Fetal and neonatal investigations (HLGT)<br>Neonatal and perinatal conditions (HLGT)<br>Neonatal respiratory disorders (HLGT)<br>Exposures associated with pregnancy, delivery and lactation (HLT)<br>Fetal therapeutic procedures (HLT)<br>Induced abortions (HLT)<br>Obstetric therapeutic procedures (HLT)
**Number of reports from VigiBase extraction**	9,346 deduplicated cases match your search	

eTable 1: Details of VigiBase initial query and MedDRA terms used for the identification of reports.

© 2023 Gougis P et al. *JAMA Network Open.*

eTable 2. Terms Corrected

MedDRA preferred terms not always associated with pregnancy	
**MedDRA preferred term**	**mapped as**
Pelvic girdle pain	Pregnancy symptom
Morning sickness	Pregnancy symptom
Ghost pregnancy (pseudo embarazo)	Pregnancy symptom
Bronchopulmonary dysplasia	Neonatal hypoxic conditions
Brief resolved unexplained event	Neonatal hypoxic conditions

MedDRA mapping problem	
**reported term**	**mapped as**
Utero, contracciones	Uterine contractions during pregnancy*

Term wrongly encoded	
**reported term**	**mapped as**
10049058	HELLP syndrome**
Cervical dilatation	translation problem, cervical dilatation for a neck edema

Terms wrongly associated with anticancer drugs	
**term**	**mapped as**
MK-8962<br>MK-8328<br>Mk-8415<br>Mk-9384	Pembrolizumab

eTable 2: Details of VigiBase initial query and MedDRA terms used for the identification of reports.

*cases of uterus contractions without pregnancy

**Italian reports link this term to HELLP syndrome, although none are linked to a pregnancy

© 2023 Gougis P et al. *JAMA Network Open.*

eTable 3. MedDRA Preferred Terms Used to Qualify Reports' Exposure Type

Exposure type	Preferred Terms
**exposure during pregnancy**	Exposure during pregnancy
	First trimester pregnancy
	Foetal exposure during delivery
	Foetal exposure during pregnancy
	High risk pregnancy
	Maternal exposure during delivery
	Maternal exposure during pregnancy
	Pregnancy
	Pregnancy on contraceptive
	Pregnancy on oral contraceptive
	Pregnancy with advanced maternal age
	Pregnancy with contraceptive device
	Pregnancy with injectable contraceptive
	Unintended pregnancy
	Unwanted pregnancy
**exposure before pregnancy**	Drug exposure before pregnancy
	Maternal exposure before pregnancy
**exposure via breast milk**	Exposure via breast milk
	Maternal exposure during breast feeding
**exposure via semen**	Exposure via body fluid
	Exposure via father
	Exposure via partner
	Maternal exposure via partner during pregnancy
	Paternal drugs affecting foetus
	Paternal exposure before pregnancy
	Paternal exposure during pregnancy
	Paternal exposure timing unspecified
	Pregnancy of partner
**exposure via skin**	Accidental exposure to product
	Exposure via direct contact
	Exposure via skin contact
	Occupational exposure to product

eTable3: MedDRA preferred terms used in reports for the identification of the timing and modality of exposure

© 2023 Gougis P et al. *JAMA Network Open.*

eTable 4. Terms Deemed Not Clinically Significant

**preferred term reported deemed not clinically significant when reported alone**	**number of occurrences in the whole cohort (*n*= 3,558)**
Fetal heart rate abnormal	10
Fetal hypokinesia	9
Uterine contractions during pregnancy	8
Weight decrease neonatal	6
Poor feeding of infant	4
Large-for-date baby	3
Uterine contractions abnormal	3
Bradycardia neonatal	2
Fetal heart rate deceleration abnormality	2
Fetal heart rate disorder	2
Fetal heart rate increased	2
Postmature baby	2
Neonatal agitation	1
Fetal arrhythmia	1
Fetal heart rate decreased	1
Fetal monitoring abnormal	1
Phimosis	1
Poor neonatal weight gain	1
Fetal tachycardia	1

eTable 4: preferred terms deemed not clinically significant when reported alone.

© 2023 Gougis P et al. *JAMA Network Open.*

eTable 5. Contingency Table

	**cases**<br><br>adverse outcome present	**controls**<br><br>adverse outcome absent
**exposure** to anti-HER2	a	c
**exposure** to other anticancers	b	d

eTable 5: contingency table for reporting odds ratio analysis of each adverse outcome. The reporting odds ratio (ROR) was defined as the ratio of the odds of exposure among reported cases to the odds of exposure among non-cases. It was used to estimate the risk of a certain outcome associated with exposure conditions. We used a two-by-two contingency table to calculate the ROR and its 95% confidence interval (CI), corresponding to ad/bc, where a = exposed cases, b = exposed non-cases, c = unexposed cases, d = unexposed non-cases.

© 2023 Gougis P et al. *JAMA Network Open.*

eTable 6. Details of Multivariable Analysis and Odds Ratio for Other Parameters for the Risk of "Oligohydramnios"

variable	level	OR for the risk of oligohydramnios [CI95%]	p-value
**cancer type**	breast cancer	**reference**	
	other malignancy	0.71 [0.38-1.3]	0.27
	cancer unknown	0.81 [0.5-1.3]	0.4
**year of report**	2009 or before	**reference**	
	2010-2014	2.1 [1.1-4]	0.022
	2015-2019	0.46 [0.24-0.88]	0.016
	2020-2022	0.71 [0.36-1.4]	0.34
**country of report**	United Stated	**reference**	
	western Europe	2.1 [1.4-3.3]	0.0011
	other country	1 [0.6-1.7]	0.91
**age of patient (yo)**	29 or younger	**reference**	
	30-39	0.75 [0.42-1.3]	0.32
	40 or more	0.44 [0.14-1.2]	0.13
	age unknown	0.27 [0.15-0.46]	0.000002
**exposure group**	exposure to antiHER2	19 [12-31]	**2.50E-^{31}**
	exposure to other anticancers	**reference**	

*eTable 6: Details of multivariablee analysis with detailed odds ratio for "**oligohydramnios**". All odds ratios were calculated using the same multivariable analysis and are displayed in Figure 2 (cf. infra).*

© 2023 Gougis P et al. *JAMA Network Open.*

eFigure 1. Directed Acyclic Graph for Assessment of Confounding Factors

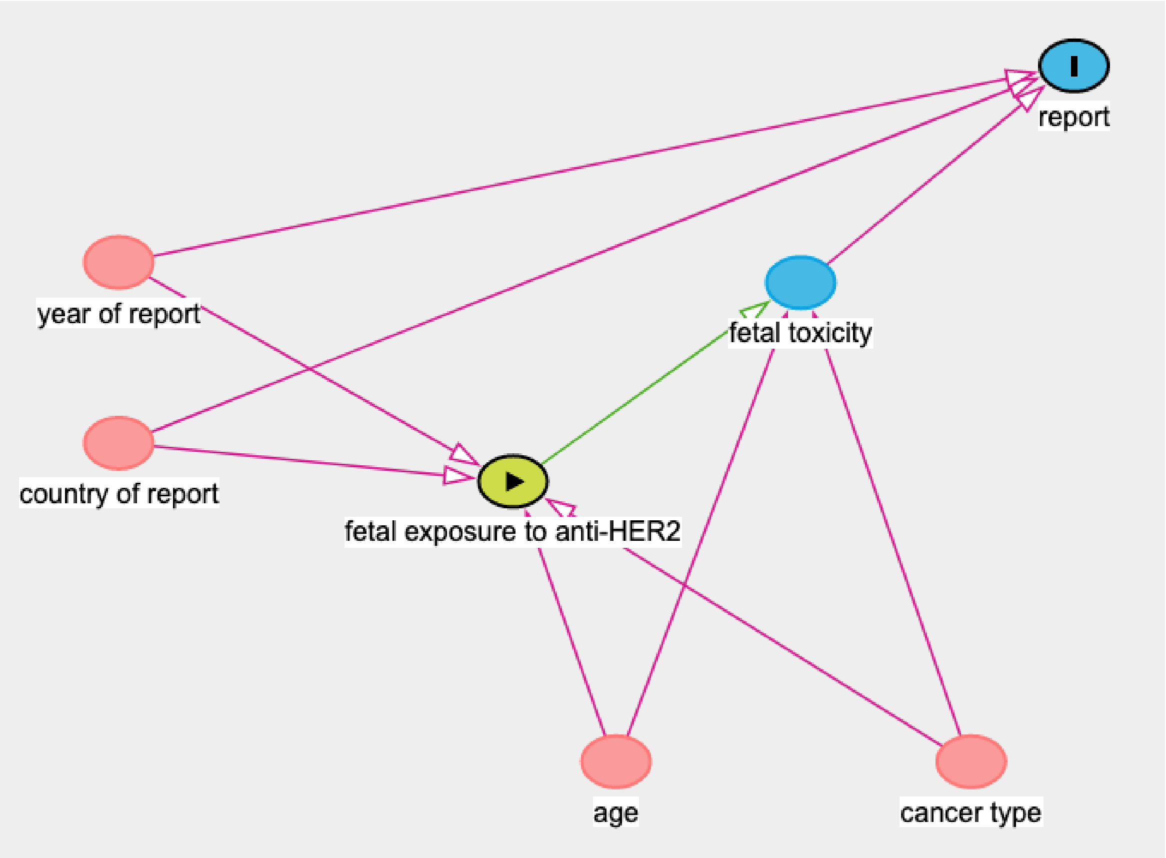

Directed Acyclic Graph for the mitigation of main confounding factors of reporting (constructed using DAGitty https://www.dagitty.net*). "▷" is the exposure and "I" is the outcome.*

© 2023 Gougis P et al. *JAMA Network Open.*

eFigure 2. Characteristics of Reports in Study Population

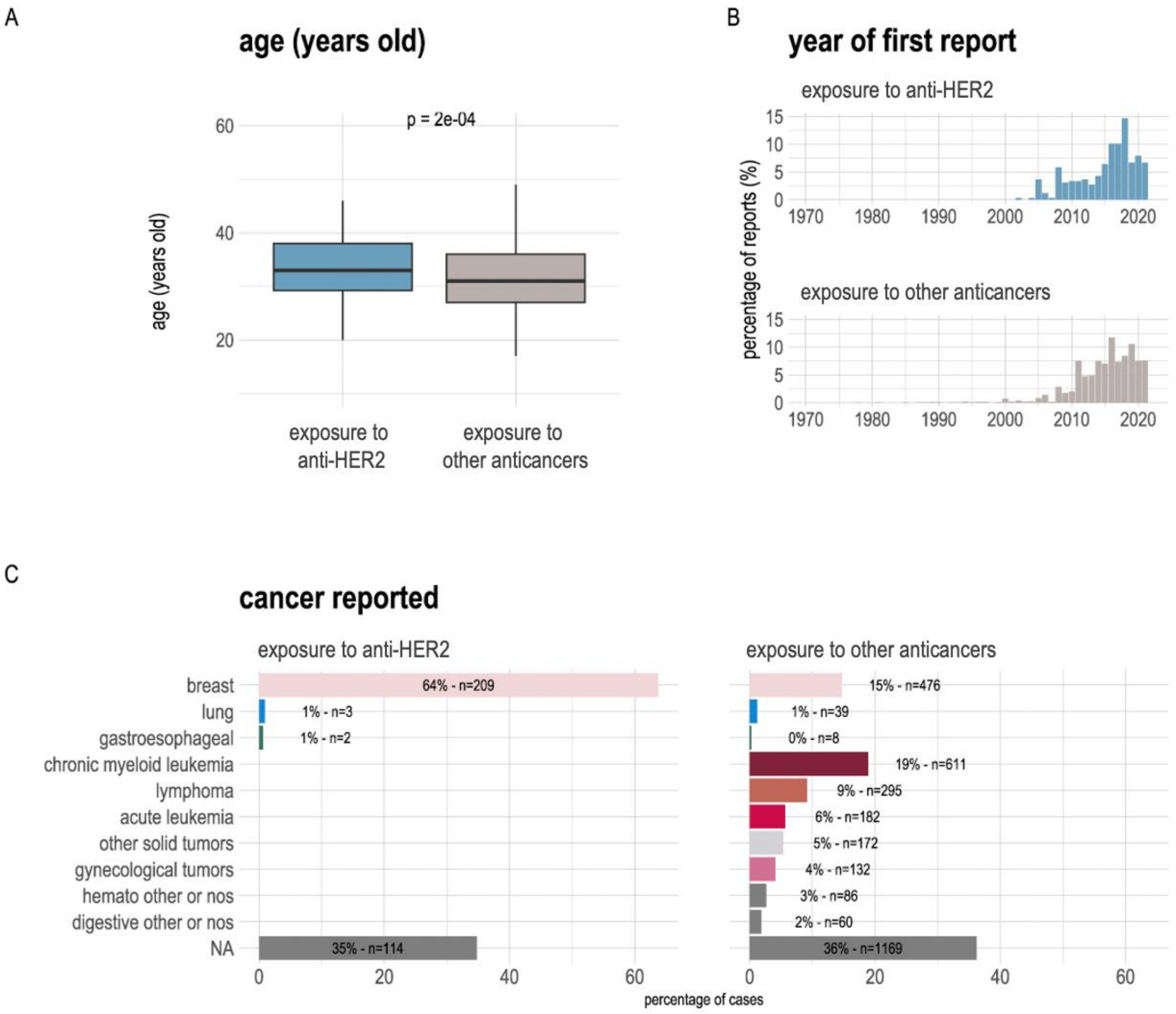

Characteristics of reports in study population for anti-HER2 exposure group compared to exposure to other anticancers. Panel A represent the age at diagnosis, panel B the year of report and panel C the cancer type identified within report.

© 2023 Gougis P et al. *JAMA Network Open.*

eFigure 3. UpSet Plot of the Reporting of Anticancer Drugs in the Anti-ERBB2-Exposed Group

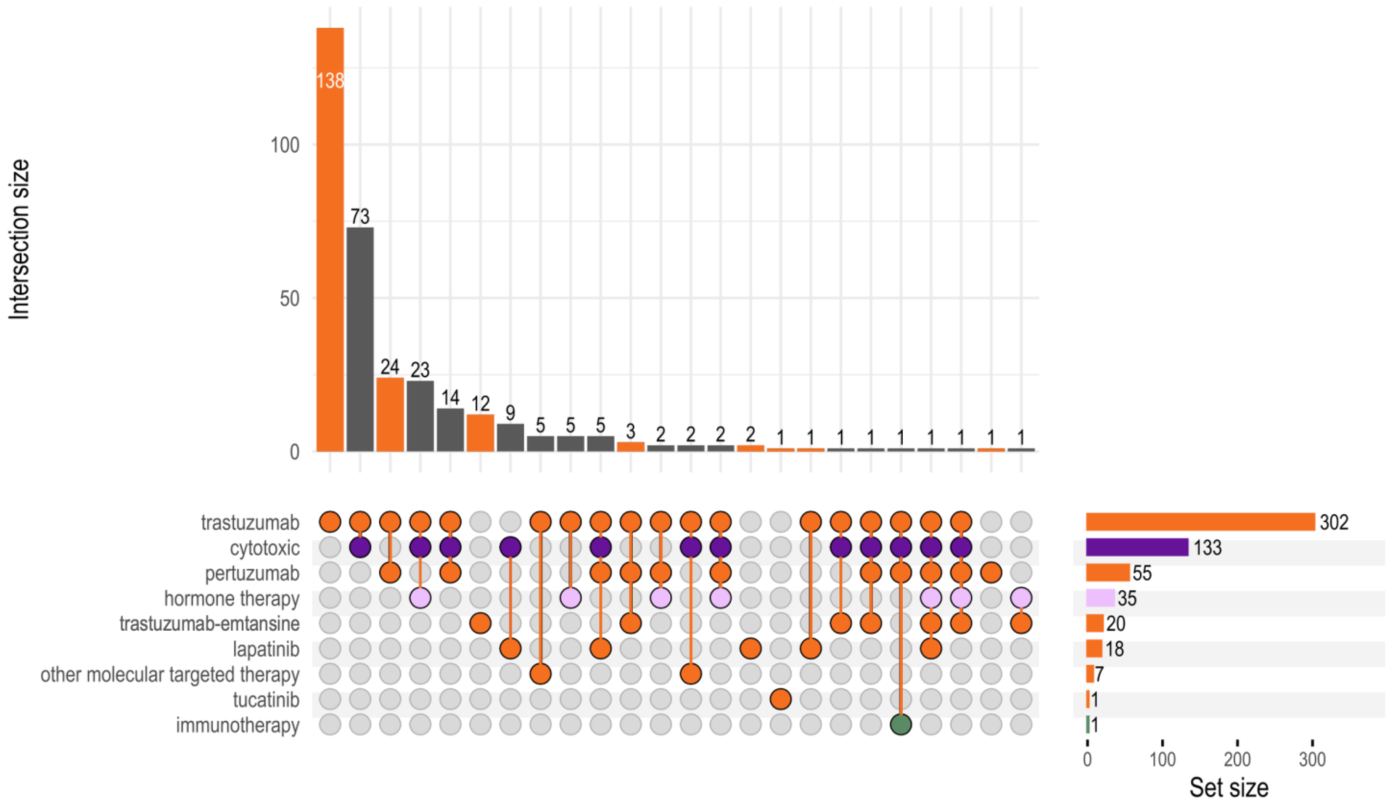

UpSet plot of the reporting of anti-HER2 with other anticancers within the anti-HER2 exposure group (n=328). The intersection size represents the number of reports for which the modality of intersection below is found. The Set size is the number of reports for which the treatment has been reported. Intersection size for reports with anti-HER2-only are in orange (total n=182), and reports with combination with other agents (cytotoxic chemotherapies, hormonotherapies, other molecular targeted therapies or immunotherapies) are displayed as gray bars (n=146).

© 2023 Gougis P et al. *JAMA Network Open.*

eFigure 4. UpSet Plot of the Co-reporting of Adverse Pregnancy and Fetal/Newborn Outcomes Overreported in Cases Exposed to Anti-ERBB2

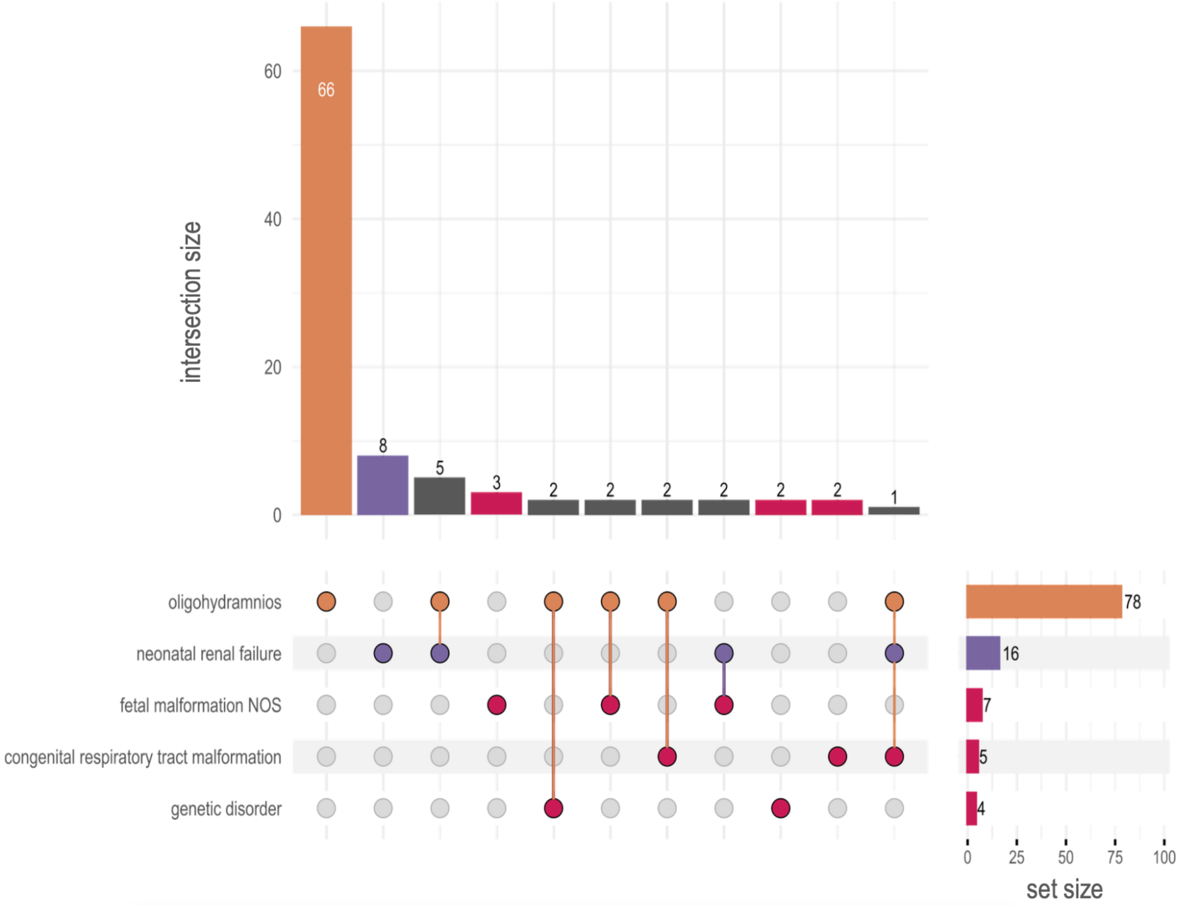

UpSet plot of the co-reporting of adverse pregnancy and fetal/newborn outcomes overreported in cases exposed to anti-HER2. Bars represent counts of pregnancy and/or fetal/newborn outcome categories in cases exposed to anti-HER2. Isolated adverse outcomes are displayed as colored bars. Combinations of adverse outcomes are displayed as gray bars. The proportion of each adverse outcome independent of combinations is displayed in the set size graph.

© 2023 Gougis P et al. *JAMA Network Open.*

eFigure 5. Disproportionality Analysis for Each Molecule

Profile of maternal and fetal/adverse outcomes by anti-HER2 molecule. The reporting odds ratio is calculated using the study population non-exposed to the molecule. NOS: not otherwise specified;

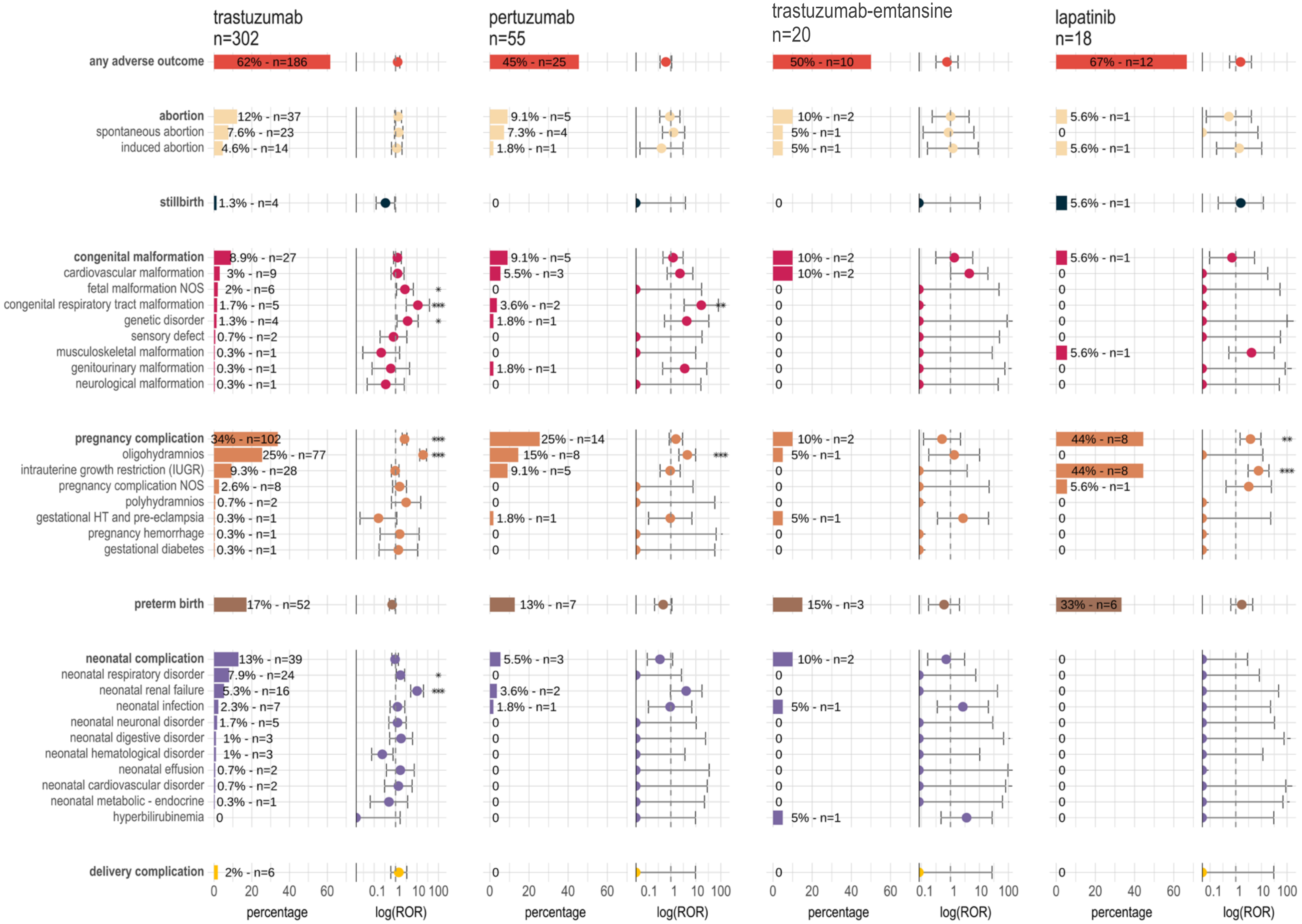

© 2023 Gougis P et al. *JAMA Network Open.*

eFigure 6. Multivariable Analysis of the Risk of Adverse Outcomes

maternal or fetal/newborn adverse outcome	Nobs anti-HER2 exposed	mulitvariate OR [CI95%]	
**any adverse outcome - n=2,018**	n=201	1.4 [1.1-1.9]	**
**abortion - n=350**	n=39	2.3 [1.5-3.6]	***
spontaneous abortion - n=205	n=24	2.5 [1.4-4.2]	***
induced abortion - n=148	n=15	1.8 [0.88-3.4]	
**stillbirth - n=129**	n=5	0.8 [0.27-1.9]	
**congenital malformation - n=270**	n=30	1.5 [0.92-2.2]	
cardiovascular malformation - n=88	n=10	1.1 [0.52-2.3]	
fetal malformation NOS - n=30	n=7	3 [1-7.6]	*
congenital respiratory tract disorder - n=10	n=5	14 [3.2-69]	***
genetic disorder - n=16	n=4	3.3 [0.78-12]	
musculoskeletal malformation - n=51	n=2	0.41 [0.066-1.4]	
sensory defect - n=29	n=2	0.95 [0.15-3.4]	
neurological malformation - n=32	n=1	0.4 [0.022-2]	
genitourinary malformation - n=19	n=1	0.39 [0.021-2.1]	
**pregnancy complication - n=632**	n=108	2.7 [2-3.5]	***
oligohydramnios - n=134	n=78	19 [12-31]	***
intrauterine growth restriction (IUGR) - n=342	n=32	1.1 [0.7-1.6]	
pregnancy complication NOS - n=65	n=9	2.7 [1.2-5.8]	*
gestational HT and pre-eclampsia - n=68	n=2	0.19 [0.029-0.65]	
polyhydramnios - n=9	n=2	3.6 [0.39-30]	
gestational diabetes - n=9	n=1	0.78 [0.037-6.8]	
pregnancy hemorrhage - n=8	n=1	2.4 [0.092-31]	
**preterm birth - n=805**	n=57	0.56 [0.4-0.76]	
**neonatal complication - n=479**	n=40	0.94 [0.63-1.4]	
neonatal respiratory disorder - n=182	n=24	1.8 [1.1-2.9]	*
neonatal renal failure - n=34	n=16	9.6 [4.2-22]	***
neonatal infection - n=69	n=7	1 [0.41-2.3]	
neonatal neuronal disorder - n=49	n=5	1.1 [0.35-2.7]	
neonatal hematological disorder - n=134	n=3	0.29 [0.07-0.8]	
neonatal digestive disorder - n=21	n=3	1.6 [0.33-5.4]	
neonatal cardiovascular disorder - n=18	n=2	1.3 [0.19-5.7]	
neonatal effusion - n=15	n=2	2.1 [0.31-9.2]	
hyperbilirubinemia - n=52	n=1	0.25 [0.014-1.2]	
neonatal metabolic - endocrine disorder - n=12	n=0	5.7e-08 [NA-3.1e+72]	
**delivery complication - n=52**	n=6	1 [0.35-2.6]	

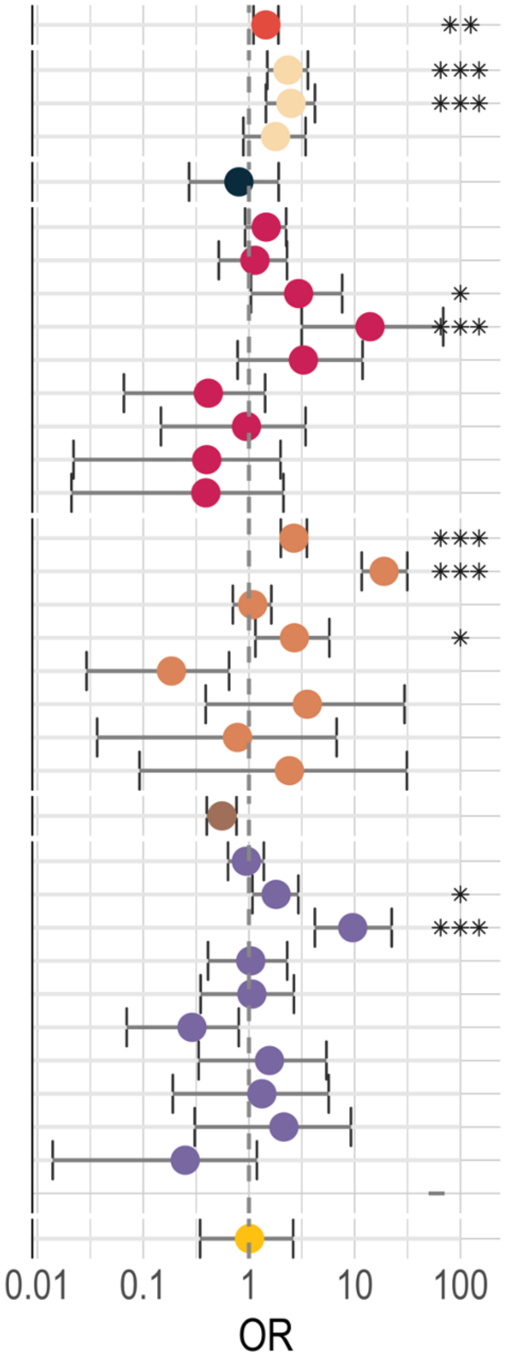

Multivariable analysis of the risk of adverse pregnancy and fetal/newborn outcomes with exposure to anti-HER2 compared to other anticancers. The adjustment was made on the year and country of the report, patient's age and cancer type. Details for oligohydramnios analysis are available in eTable 6.

Abbreviations: CI95%: 95% confidence interval; HT: hypertension; NOS: not otherwise specified; OR: odds ratio

© 2023 Gougis P et al. *JAMA Network Open.*

eFigure 7. Sensitivity Analysis Within the Subpopulation of Reports With an Identified Breast Cancer

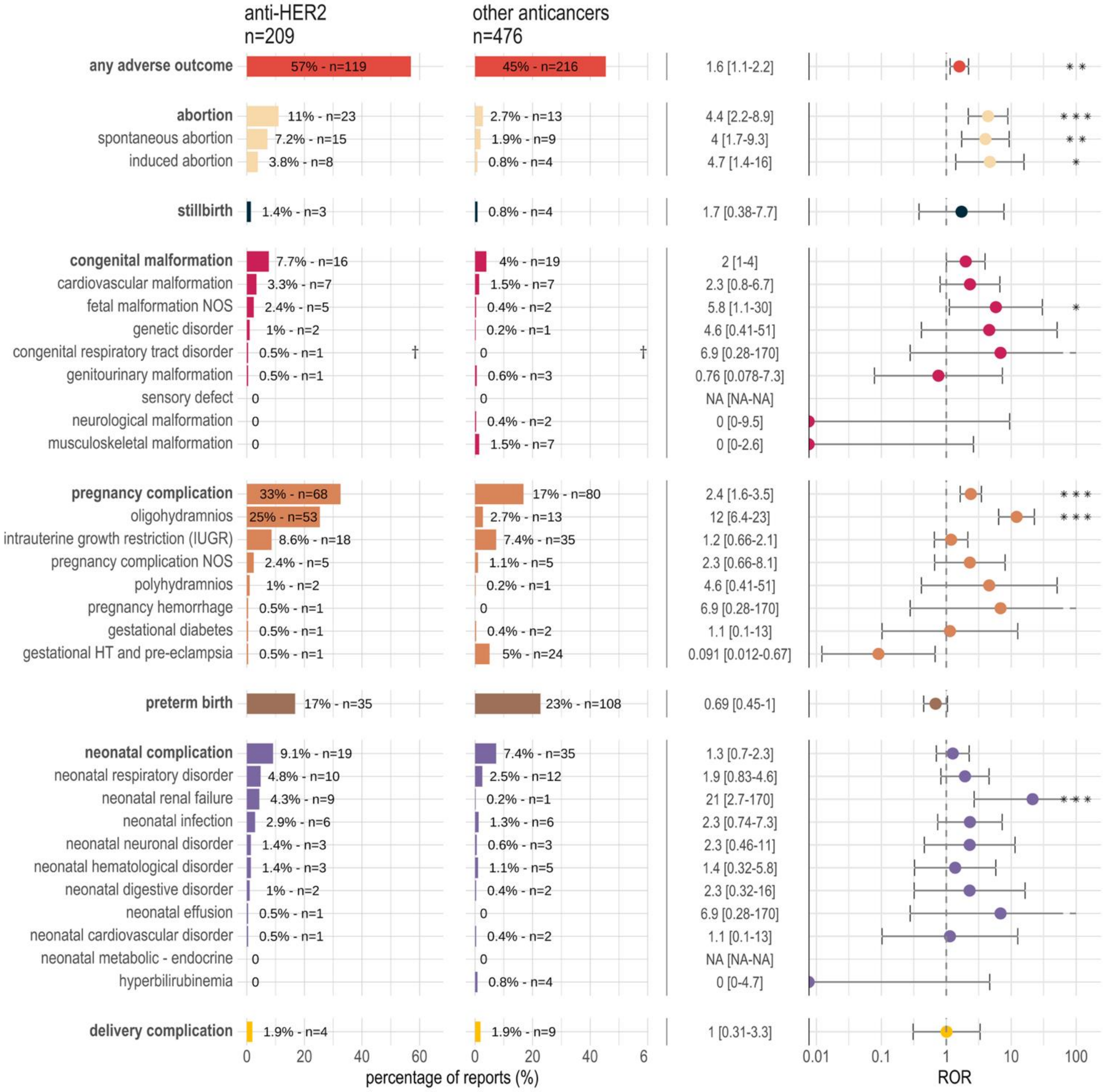

Sensitivity analysis within the subpopulation of reports for which Breast Cancer was identified. Of note, only 2% of reports with anti-HER2 and identified cancers were not Breast Cancers (cf. eFigure 2).

© 2023 Gougis P et al. *JAMA Network Open.*

eFigure 8. Sensitivity Analysis Within the Subpopulation of Reports Treated With Single-Class Drugs

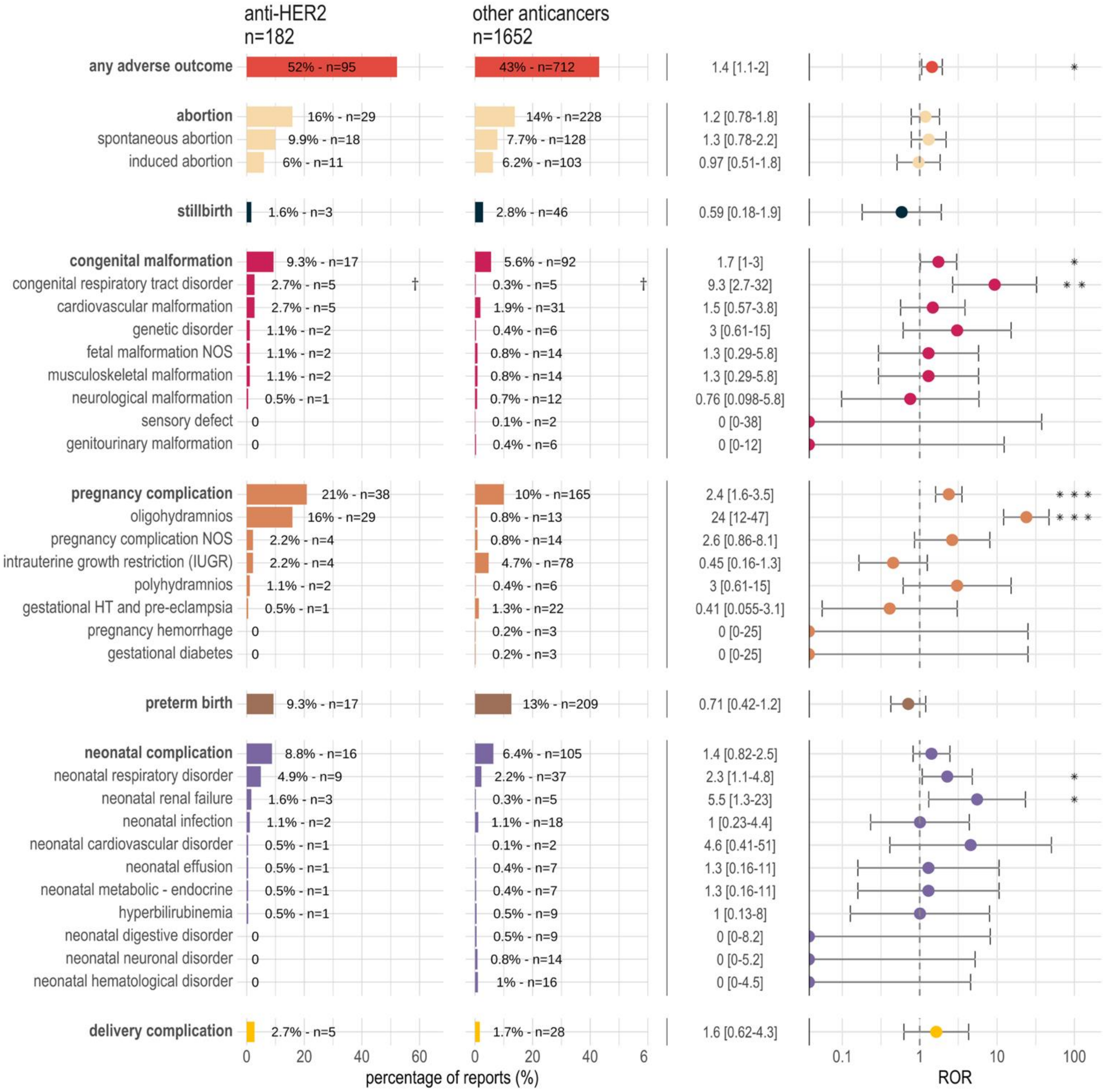

Sensitivity analysis within the subpopulation of reports treated with single-class drugs. Any reports with a combination of drug classes were excluded in both exposure groups. ROR is the reporting odds ratio for the risk of maternal and fetal/newborn adverse outcomes within the subgroup exposed to anti-HER2 compared to the exposition to other anticancers.

© 2023 Gougis P et al. *JAMA Network Open.*
