# Supplementary material for: Treatments During Pregnancy Targeting ERBB2 and Outcomes of Pregnant Individuals and Newborns
Source: JAMA Netw Open. 2023 Oct 26;6(10):e2339934. doi: 10.1001/jamanetworkopen.2023.39934 (PMC10603505; doi:10.1001/jamanetworkopen.2023.39934)
Supplement: Supplement 2. — Data Sharing Statement [file jamanetwopen-e2339934-s002.pdf]

Data Sharing Statement

Gougis. Treatments During Pregnancy Targeting ERBB2 and Outcomes of Pregnant Individuals and Newborns. *JAMA Netw Open*. Published October 26, 2023. doi:10.1001/jamanetworkopen.2023.39934

Data
Data available: No

Additional Information
Explanation for why data not available: data publicly available at https://who-umc.org/vigibase/
